# Support Vector Machine-Based Classification of Vasovagal Syncope Using Head-Up Tilt Test

**DOI:** 10.3390/biology10101029

**Published:** 2021-10-12

**Authors:** Shahadat Hussain, Zahid Raza, Giorgio Giacomini, Nandu Goswami

**Affiliations:** 1 School of Computer and Systems Sciences, Jawaharlal Nehru University, New Delhi 110067, India; shahad96_scs@jnu.ac.in; 2 General Hospital, 8720 Knittelfeld, Austria; med.juk@kages.at; 3 Otto Loewi Research Center for Vascular Biology, Immunology and Inflammation, Medical University of Graz, 8036 Graz, Austria; nandu.goswami@medunigraz.at; 4 Alma Mater Europaea, 17 2000 Maribor, Slovenia

**Keywords:** support vector machine, neuro-mediated syncope, classification, machine learning, head-up tilt (HUT) test

## Abstract

**Simple Summary:**

Syncope is a medical condition triggered by short-lived interruption of the oxygen supply to the brain, which may result in free fall or accidents. The diagnosis of syncope is a challenging task, as various other states of altered consciousness present with the same symptoms as syncope. This work uses historical medical data for the diagnosis of syncope using sophisticated computing solutions. The experimental results prove the effectiveness of the approach, leading to the proactive prediction of syncope.

**Abstract:**

Syncope is the medical condition of loss of consciousness triggered by the momentary cessation of blood flow to the brain. Machine learning techniques have been established to be very effective way to address such problems, where a class label is predicted for given input data. This work presents a Support Vector Machine (SVM) based classification of neuro-mediated syncope evaluated using train–test–split and K-fold cross-validation methods using the patient’s physiological data collected through the Head-up Tilt Test in pure clinical settings. The performance of the model has been analyzed over standard statistical performance indices. The experimental results prove the effectiveness of using SVM-based classification for the proactive diagnosis of syncope.

## 1. Introduction

Syncope is a medical condition resulting in a transient loss of consciousness (LOC) or postural tone with spontaneous recovery. A short-lived interruption of the oxygen supply to the brain is the most fundamental aspect of the induction of syncope [1]. Depending on various underlying conditions of its occurrence, syncope is primarily classified into three categories: vasovagal, cardiovascular and orthostatic hypotension (OH) [2]. The cardiovascular and OH forms of syncope, found among older adults, primarily happen due to the various health conditions involving the circulatory system and cardiac dysfunction. The occurrence of these episodes of syncope is life-threatening and thus requires serious medical attention. Vasovagal or neurally mediated syncope, found in young adults, is most common form of syncope, which primarily happens due to a quick transient drop in the systemic arterial Blood Pressure (BP) required for the sustenance of cerebral perfusion. Usually the drop in Heart Rate (HR) is the triggering phenomenon leading to the drop in systemic arterial BP and syncope. The product of cardiac output (CO) and total peripheral resistance (TPR) provides the measure of systemic arterial BP. Any significant decline in CO or TPR has the potential to create cessation in the cerebral blood flow and, consequently, global cerebral hypoperfusion [3]. Though not life-threatening in nature, this form of syncope has a great impact on the quality of life and handling of other health conditions alongside. Besides, it prompts the loss of postural tone, which sometimes leads to falls or accidents causing serious harm to the body. The diagnosis of vasovagal syncope in itself is a challenging task as various other states of altered consciousness require a different path of treatment and expertise. Thus, evaluating patients with loss of consciousness (LOC) or near LOC and establishing a true form syncope is a crucial step in the treatment process [4].

The use of high-end computing solutions at this crucial stage of diagnosis is anticipated to add great benefits for resource-constrained healthcare organizations. Healthcare 4.0, by the usage of Artificial Intelligence (AI) and Machine Learning (ML) coupled with the Internet of Things (IoT) and Big Data, is facilitating a refined diagnostic and treatment procedure and thus provides a significant gain in the efficiency of and cost-saving for healthcare services [5,6,7]. Machine learning is the process whereby a computer manipulates a suitable statistical model utilizing observed data to generate an outcome or classify observations about new data. The objective of ML is to develop capabilities into data driven machines by enabling advanced algorithms and statistical methods to achieve more powerful predictions compared to a rule-based system. ML models are extensively being used to compute valuable predictions in various domains including robotics, finance, retail, transport and healthcare, etc. Depending on the desired outcome and the characteristics of the data in question, ML models are broadly classified into three categories viz. supervised, unsupervised and reinforced ML. Supervised ML trains the models involving labelled data and make predictions about new data using the same information. Unsupervised ML trains models involving data whose labels are not known and make clusters of similar data based on the hidden patterns in them. Similarly, reinforced ML iteratively improves its performance by getting feedback from environments in the forms of rewards or penalties for the actions it performs. The availability of an enormous quantity of patient-related data in the form of Electronic Health Records (EHR) has created new opportunities for researchers, enabling high-grade classifications, predictions and pattern recognitions using large volumes of high dimensional data and fueling advances in both the science and practice of medicine [8,9].

The objective of this paper is to classify syncope and non-syncope events of patients using the supervised machine learning algorithm Support Vector Machine (SVM) applied to the patients’ true physiological data, which were collected through HUT tests, to provide the differentiation between instances of syncope and non-syncope. Considering the volume and dimension of the collected data, SVM qualifies as a suitable classifier because it can efficiently discriminate entities containing n-dimensional vectors. The raw data recorded using the HUT test are first refined by some basic statistical methods before being consumed by the SVM classification model. The results derived by the model are compared with the results of the k-nearest neighbors (KNN) and stochastic gradient descent (SGD) models employed to the same dataset and in similar computing environments. The models are adjudicated over various performance measures of *accuracy*, *precision*, *recall*, *F1-Score* and area under the curve of Receiver Operating Characteristics (*AUC-ROC*).

The remainder of the paper is organized as follows: Section 2 discusses the limitations and inadequacies of the existing work reported in the literature. Section 3 presents the details of the working of the model in its various phases, including procedures of data collection, data organization and data preparation leading to an SVM-based classification of syncope and its performance in comparison with KNN and SGD-based models. Section 4 presents the limitations of the work while stating possible future directions. This is followed by Section 5, which concludes the work.

## 2. Related Work

The classification between syncope and non-syncopal events based on true physiological data has scarcely been touched upon. This section touches upon some of the work, though limited, relating to syncope. The use of a Random Forest Classifier (RFC) to classify events of syncope and non-syncope was reported in [10]. However, the work does not address the very important issue of handling the imbalance of data for the considered cases of syncope and non-syncope, which becomes a limiting factor. Various classification works based on the observations of individual physicians have been reported [11,12,13,14]. Since the works are based on the laboratory findings of individual physicians, the findings become phenomenological and lack objectivity. The work in [15] differentiates the cases of syncope from other forms of loss of consciousness, but the dataset generated for the work is based on responses to a questionnaire instead of on the analysis of true physiological data, e.g., heart rate and beat-to-beat recording of blood pressure. The early prediction of syncope using the HUT test was reported in [16]. However, the work only considers the amplitude of systolic BP and dynamic interaction between two successive R-waves of the QRS signal on the electrocardiogram (RR-interval) as the differentiating parameters.

The work presented in this paper gains significance due to the fact that it is based on the true physiological data collected through the HUT test. The dataset utilized for the work has been balanced with an equal number of instances of syncope and non-syncope, which results in better training and hence better classification. The results of the analysis presented in the work are therefore more reliable, as they have been adjudicated through the statistical indices of various measures of performance.

## 3. Syncope Classification Model

The foundations of this work are based on two central hypotheses:

**Hypothesis** **1** **(H1).** *Etiology of syncope can be derived by beat-to-beat examination of BP along with continuous analysis of HR variability, and;*

**Hypothesis** **2** **(H2).** *Mathematical modeling and machine learning algorithms can provide a near-accurate diagnosis for patients having syncopal episodes correlated with autonomic dysfunction.*

**Figure 1:**
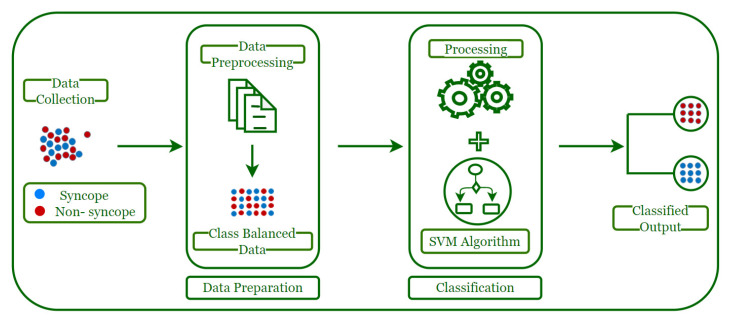
Flow Diagram of the Working of Model.

The workings of the model can be summarized with the help of a diagram, as shown in Figure 1, which presents the various steps followed by the model.

The process has been divided into four stages. The first stage corresponds to the data collection which is the input dataset for the model. The input data generated are observed to be skewed, with only some cases reported with syncope. If such data are used as is, this may result in a scenario in which the training data will have very few minority class instances (syncope) and a very large number of majority class instances (non-syncope). This results in the machine being trained inefficiently, leading to poor predictive performance. Accordingly, in the data preparation stage, the imbalance in the input data is addressed by converting the data into a balanced set, which is an essential step for any machine learning classifier (including SVM) to work properly. The output of this stage results in a class-balanced dataset which is employed by the Support Vector Machine (SVM) algorithm for processing to generate the classified data which are used for predicting syncope. These stages are elaborated on in the following sections.

### 3.1. Data Collection

The data utilized for this research were obtained from patients undergoing routine tilt table testing at the Syncope Clinic, General Hospital (LKH), Knittelfeld, Austria. The data were collected from a total of 687 patients in a purely clinical setting. All these patients had histories of syncope or dizziness upon standing up. Accordingly, the patients having recurrent syncope, or who were supposed to be high-risk patients having experienced at least a single episode of syncope, were considered for the study. All participants provided their written informed consent.

After arriving at the hospital, the patients were equipped with BP and electrocardiographic sensors. Data recorded through the sensors were saved digitally with the help of analog-to-digital converters communicating with computers. Specifically, hemodynamic responses. such as Heart Rate (HR) and mean arterial pressure responses at baseline and at the development of orthostatic intolerance during tilt table testing, were measured. The inclusion and exclusion criteria for patients undergoing tilt table testing were strictly followed. Further, in this exploratory study, continuous and non-invasive beat-to-beat HR and BP measurements were recorded.

**Figure 2:**
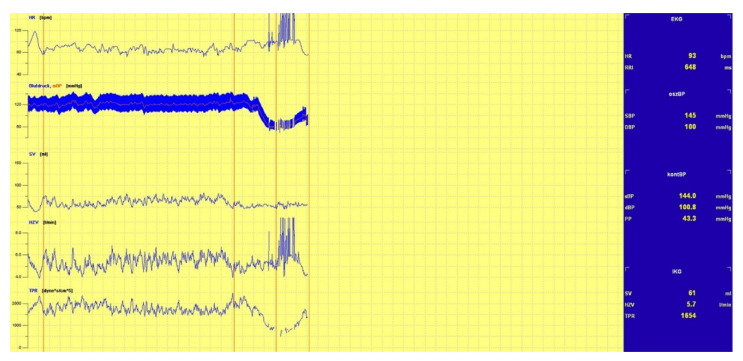
Drop in BP.

**Figure 3:**
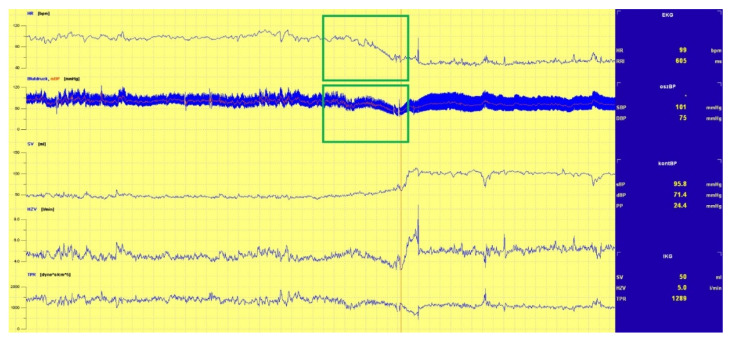
Drop in both BP and HR.

**Figure 4:**
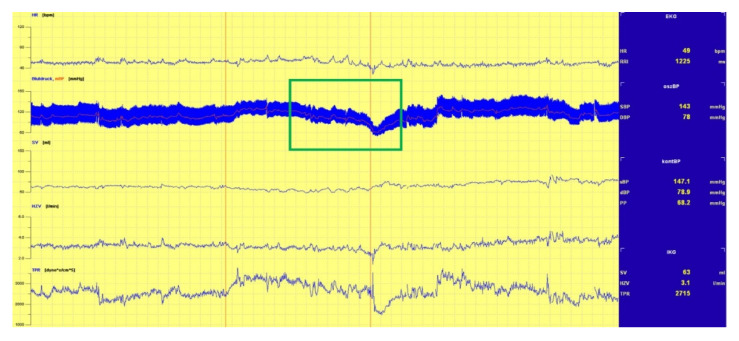
Continuous Drop in BP.

The tests for this study found three main underlying mechanisms responsible for the triggering of the induction of syncope:

(1) A sudden drop in BP, as shown in Figure 2;
(2) A drop in HR, and thus drop in BP, as shown in Figure 3, and;
(3) A continual drop in BP, as shown in Figure 4.

#### 3.1.1. Head-Up Tilt (HUT) Test

A footplate-supported table equipped with an automatic tilting mechanism was used for the HUT test. Before the tilting of the table, patients were observed in a supine position for ten minutes. The flat-top bedding surface containing safety straps was then tilted to the angles of 60° to 80° in a quick span of time. The rationale behind the whole action is that a sudden change in posture sometimes induces vasovagal syncope, which is characterized by a sudden drop in HR and BP [17]. Constant monitoring of electrocardiographic signals, along with continuous beat-to-beat checking of BP, was performed during the test.

The data were collected using the Task Force Monitor (CNSystems, Graz, Austria). All data obtained were obtained from each of the 3 positions: supine–HUT–return to supine and then the data were averaged. Systolic blood pressure and diastolic blood pressure were measured at the right brachial artery using the oscillometric method. Hemodynamic parameters such as stroke volume (SV), cardiac output, total peripheral resistance were recorded beat-to-beat using impedance cardiography [18,19,20]. Total peripheral resistance was calculated from the CO and BP values measured with a finger sensor; automatic calibration was performed using the oscillometric method. Heart rate measurements were carried out using RR-interval.

Presyncope is the state immediately preceding a syncopal event, defined as a sudden, brief, transient loss of consciousness [21,22,23]. ‘Physiological’ syncope during orthostatic loading develops as the result of critically diminished cardiac preload due to low venous return. Once the brain perfusion is reduced to below a critical level, a “vasovagal attack” is triggered, which leads to decreases in heart rate and blood pressure and sudden dilation of the arterial vessels, leading to the loss of consciousness. The following criteria were used for presyncope: heart rate decreases by ≥15 bpm or blood pressure decreases to less than systolic 80 mmHg or by ≥25 mmHg/min; diastolic decreases by ≥15 mmHg/min; and/or nausea, cold-clammy skin or dizziness [24,25].

Tests concluded with the finding that out of 687 patients, 96 were recognized to have an induction of syncope while remaining 591 patients were able to keep control of their BP and HR, falling in the category of non-syncope. Table 1 presents the distribution of the patients having syncope in terms of age group and gender.

#### 3.1.2. Data Organization

The BP and HR data recorded in the test were grouped as Beatstats, Cardiacbeatstats, HRVstats, dBPVstats and sBPVstats, as shown in Table 2. A total of 48 different physiological conditions against each subject were recorded in the proper format. It is beyond the scope of this work to provide a complete description of all the physiological indicators; however, a summary of each parameter, along with their quantifying mechanisms and their units of measurement, has been presented.

### 3.2. Data Preparation

The raw data recorded during tests consists of the continuous observation of HR and beat-to-beat checking of BP, which needs to be discretized for better applicability on ML models. TFM employed during the tests facilitates the discrete values of the continuous health indicators required for the classifications. To prepare the final dataset, the discrete values of the health indicators are further preprocessed with basic statistical functions of the maximum value (max), minimum value (min), mean (mean), standard deviation (sd), variance (var), coefficient of variance (vc) and standard error of the mean (sem).

It was observed that the data generated by the HUT test result in an imbalanced dataset, as only 96 patients were found to have induction of syncope against a total of 687 patients in the study. This imbalance in the data could result in a bias towards the majority class. The imbalance in the dataset can be resolved either by assigning different class weights for the majority and minority classes, or by oversampling the minority class with artificially created instances. The suitability of the method to be used depends on the considered classification models and the dataset. Methods such as Synthetic Minority Oversampling Techniques (SMOTE) [26] and Adaptive Synthetic (ADASYN) [27] have been reported in the literature for the generation of synthetic data. This work uses SMOTE to create the artificial instances of the minority class to address the data imbalance considering that the use of SVM-based model supported by the fact that KNN model used in the comparative analysis exhibits better suitability towards SMOTE.

#### Principal Components Analysis

**Figure 5:**
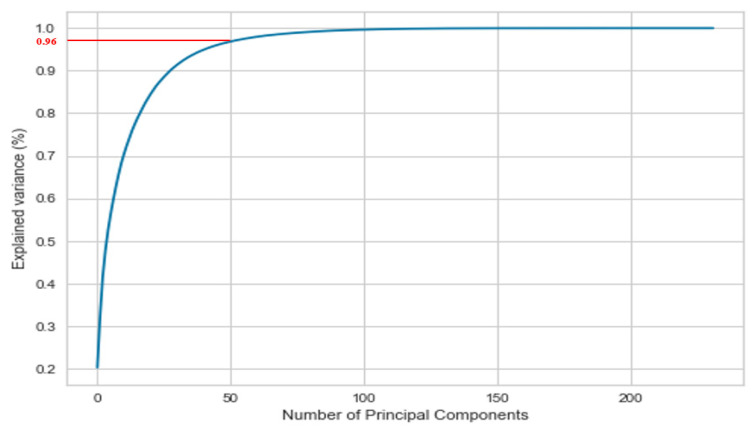
Progression of Explained Variance with PC.

To provide a view into the correlations and patterns between features of the dataset, principal component analysis (PCA) was performed on it in line with the work reported in [28]. Table 3 presents the variance explained by the significant principal components. Accordingly, the progression of cumulative variance explained by the principal components (PCs) is presented in Figure 5.

**Figure 6:**
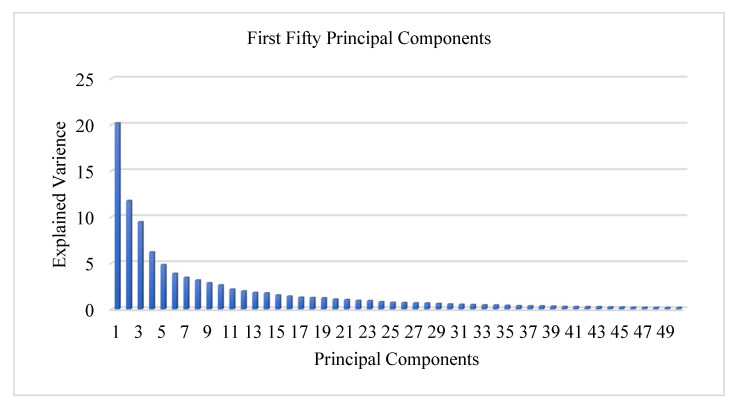
Variance Explained by First Fifty PCs.

It can be inferred from Table 3 and Figure 5 that first PC explains 20.17% of the variance in the dataset, first two PCs explain 31.95% of the variance in the dataset, the first three PCs explain 41.41% of the variance and so on. As can be seen, the first fifty PCs contribute almost 96.71% of the variance of the data, while remaining 182 PCs contribute only 03.29% of it. Figure 6 depicts the individual contributions of the fifty PCs contributing towards the 96.71% of the overall variance of the data.

**Figure 7:**
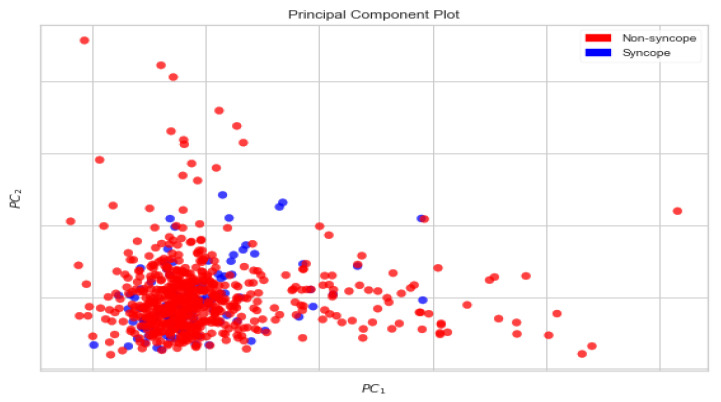
2-Dimensional Plot of First Two PCs.

The individual contributions of the first 50 PCs have been presented in Figure 6. As can be seen, the first PC (PC-1) contributes 20.17% of the variance, the second PC (PC-2) contributes 11.77% of the variance and the third PC (PC-3) contributes 09.45% of the variance, and so on and so forth. The scatter plot shown in Figure 7 depicts the variance explained by the first two PCs (PC-1 and PC-2) in a 2-dimensional space.

**Figure 8:**
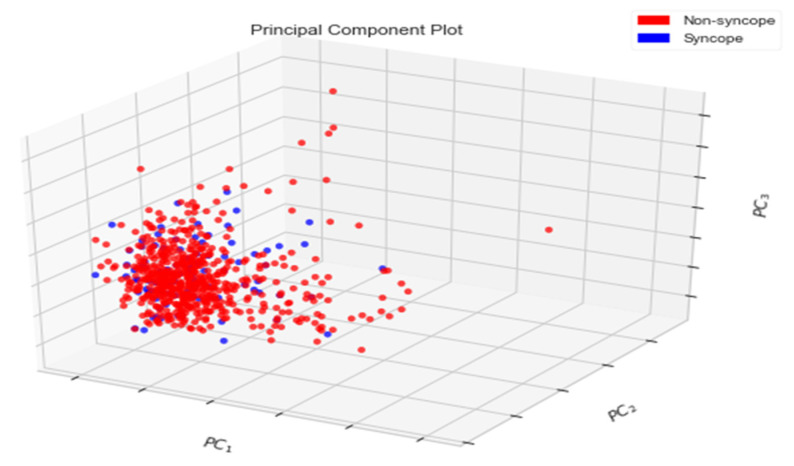
Three-Dimensional Plot of First Three PCs.

Figure 7 shows that the classes of syncope and non-syncope are not well separated from each other, as PC-1 and PC-2 together explain only 31.94% of the total variance. Similarly, an instance of the scatter plot shown in Figure 8 depicts the variance contained by first three PCs (PC-1, PC-2 and PC-3) in a 3-dimensional space.

Again, it can be inferred from Figure 8 that the classes of syncope and non-syncope are not well separated, as PC-1, PC-2 and PC-3 together explain only 41.40% of the variance.

### 3.3. Data Classification

This stage corresponds to the use of machine learning algorithm, which is SVM, in this case, to be applied to the data collected which has been collected and pre-processed in the previous stages. This is the most important stage of the process which determines the utility of the work in meeting the classification objectives.

#### 3.3.1. Support Vector Machine

Machine learning offers the use of many classification algorithms, each having its own advantages and drawbacks. Which algorithm will work well in a given case depends on the type of the problem and the dataset in question. In general, it has been established that the performance of the machine learning algorithms can be ascertained only through trial and error and through performance metrics.

In the context of this work, there were several factors which were taken into account for considering SVM-based classification of syncope. SVM generally does not suffer from the condition of overfitting and performs very well when there is a clear indication of separation between classes. It shows better adaptability towards data that are not regularly distributed and have an unknown distribution. The kernel of SVM provides non-parametric functions that allow the choosing of non-linear functions depending on the data being operated on and thus performs complex classifications with better results than the other classifiers. Further, outliers have less influence over SVM compared to other classifiers, providing fewer chances for the results to be skewed. Compared to other classifiers, SVM derives better results in quicker span of time. In addition, using the kernel functions of SVM the, input data can be converted into high dimensional data, avoiding the need for linearly separable data, which are required by other classifiers. The data considered in this work contain 231 attributes against each patient, which were derived from the 48 physiological indicators listed in Table 2. These data can be considered high dimensional, thus making the suitable for use with SVM. To summarize, the ability to deliver unique solutions makes SVM a robust model for this task compared to other models where more than one solution can be generated corresponding to each local minimum.

A support vector machine (SVM) is a linear classifier that works on margin optimization principles [29]. It performs the classification task by creating a hyperplane in a higher-dimensional space that optimally splits the data into two groups. For a dataset having *m* given training examples $\{\left(x_{1}{,y}_{1}\right),\ldots ,\left(x_{m}{,y}_{m}\right)$}, where $x_{i}$∈ R^N^ and $y_{m}$∈ {−1, 1}, SVM training tries to create the optimal hyperplane by evaluating the weight *w* and bias *b* for the linear decision function *f*(*x*) = *w*·*x* + *b*. The closest distance from the datapoints to the decision boundary is called the margin. For two oppositely margined data points *x1*, *x2* with *f*(*x1*) = 1 and *f*(*x2*) = −1, the margins can be evaluated as shown in Equation (1);

[*f*(*x_1_*) − *f*(*x_2_*)]/||*w*|| = *2*/||*w*|| (1)

In order to find the optimal hyperplane, SVM solves the optimization problem which is given in Equation (2);

*min* (||*w*||/*2*) (2)

*s.t*$y_{i}$*(w^T^x_i_ + b) ≥ 1*, where *i* = 1, 2,…, *m* and *w^T^* denotes the transpose of *w*.

As maximization of *2/||w||* is equivalent to minimization of *||w||/2*, the optimization problem can be transformed into its dual problem that gives the quadratic problem as presented in Equation (3);

(3) $$\max\;\sum_{i=1}^{m}\alpha_{i}-\frac{1}{2}\sum_{i,j=1}^{m}\alpha_{i}{\;\alpha }_{j}\;y_{i}\;y_{j}(x_{i}\cdot x_{j})$$

*s.t* $\sum_{i=1}^{m}\alpha_{i}{\;y}_{i}$ = 0; *α* ≥ 0 ∀i = 1,…, *m*.

While solving the problem for optimal hyperplane it gives the parameter *w*, which is calculated as shown in Equation (4) as

(4) $$w=\sum_{i=1}^{m}\alpha_{i}{\;y}_{i}\;x_{i}$$

Thus, the linear decision function *f*(*x*) in dual space is evaluated as shown in Equations (5) and (6);

(5) $$f(x)=\sum_{i=1}^{m}y_{i}\alpha_{i}{(x}_{i}\cdot \;x)+b,\;\mathrm{where}\;b\mathrm{can}\;\mathrm{be}\;\mathrm{evaluated}\;\mathrm{as}$$

(6) $$b=-½\;[\max_{y=-1\;}(w\;\cdot \;x_{i})+\min_{y=1}\;(w\;\cdot \;x_{i})]$$

The Karush–Kuhn–Tucker theory demonstrates that the examples satisfying the condition of $y_{i}$(*wx_i_* *+ b*) = 1, are the resultant non-zero instances.

#### 3.3.2. Performance Metrics

Evaluating the performance of the classifier is an essential part of any machine learning model, as it delineates the correctness of the classification. For a classification problem of two or more output classes, the Confusion Matrix is one of the most intuitive metrics for finding the correctness of the model [30]. A confusion matrix is a square matrix having *C_i j_* as the data elements, where *i* and *j* denote the true label and predicted label of the data group, respectively. For a binary output classification problem, the confusion matrix is a 2x2 matrix of four elements viz. *C_00_, C_01_, C_10_ and C_11_*, as shown in Table 4.

Using the above confusion matrix, any classification algorithm can be compared based on five performance measures viz. *accuracy*, *Precision*, *Recall*, *F-1 Score* and *AUC-ROC*. In general, accuracy is a simple and effective measure to judge the performance of a ML model. However, it is not a reliable metric for an imbalanced dataset. To address the concern of biasness towards the majority class in an imbalanced dataset, which can result in possible false accuracy, other performance metrics such as *precision*, *recall*, *F1-Score* and *AUC-ROC* are used. These parameters are briefly discussed below [31,32].

*Accuracy*: This is defined as the ratio of correct predictions to the total predictions made by the model. It is evaluated using the elements of the confusion matrix as shown in Equation (7);

(7) $$Accuracy=\frac{C_{00}{+C}_{11}}{C_{00}{+C}_{01}{+C}_{10\;}{+C}_{11}}$$

*Accuracy* is a measure of the effectiveness of the machine learning model in establishing the relationship between the parameters and making correct classifications.

*Precision*: This is defined as the ratio of true positive predictions to the total number of positive predictions. *Precision* is a measure of the relevancy of the results and is calculated as shown in Equation (8);

(8) $$Precision=\frac{C_{11}}{C_{01}{+C}_{11}}$$

*Recall*: This is evaluated as the ratio of true positive predictions to actual positive samples. *Recall* is a measure of the total number of predictive instances correctly classified by the model, which is calculated as shown in Equation (9).

(9) $$Recall=\frac{C_{11}}{C_{10}+C_{11}}$$

For binary classifications, *Recall* is also referred to as the sensitivity of the model.

*F1-Score*: This is the harmonic mean of precision and recall that is used to create the balance between the false positives and false negatives of the samples. It is evaluated as shown in Equation (10);

(10) $$F-Score=2\;\times \;\frac{Precision\;\times \;Recall}{\begin{array}{c} Precision+Recall\\ . \end{array}}$$

A good *F1-Score* proves the effectiveness of the model and indicates that the model is exhibiting low false positives and low false negatives.

*AUC-ROC*: The receiver operating characteristics (ROC) curve is a technique to depicts the visualization, organization and selection of classifiers for all the classification thresholds. The ROC curve is plotted between the true positive rate on the y-axis and the false-positive rate on the x-axis. The area under the ROC curve (*AUC-ROC*) is the significant measure of the performance of binary classifiers.

### 3.4. Classified Output

A simulation was carried out for using the SVM model and evaluating the results on the basis of various performance indices. The hardware, software and API specifications used in the study are listed in Table 5.

As mentioned in Section 2, in general, the reported works in the domain lack the following:

The imbalance in classes of data has not been addressed;
The generated dataset is based on responses to a questionnaire instead of the true physiological data;
The generated dataset is based on the observations made by individual physicians and not on continuous observation of the heart rate and beat-to-beat recording of blood pressure.

In the light of the above, it was not possible to compare the results found here with similar works. However, based on the application of the classification model to the dataset, and to provide a better look into its consistency, the evaluation of the proposed model was performed in two ways viz. The Train–Test–Split evaluation and *K*-fold cross-validation evaluation. Additionally, the results of the SVM-based classification model were compared with two models viz. k-Nearest Neighbor (KNN) [33] and Stochastic Gradient Descent (SGD) learning [34] using the same dataset in the same computing environment.

The SVM model-related parameters considered for experimentation are: *kernal*, *gamma* and *C*. The mathematical functions used by SVM algorithms are known as the *kernel*. Parameter ‘*C*’ controls the tradeoff between margin maximization and error minimization and is a measure used to avoid misclassification. The parameter *gamma* is used to address the variance in the model. Using Scikit learn, *kernel* has been set as linear, *C = 2* and *gamma = auto* decided based on standard practices. The other considered parameters for simulating the SVM, SGD and KNN algorithms are listed in Table 6.

#### 3.4.1. Contributing Features

**Figure 9:**
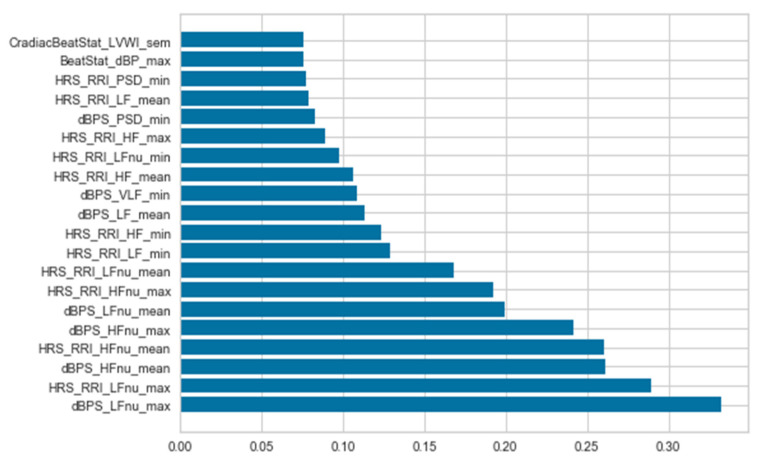
First Twenty Most Contributing Features of the Data.

The dataset has 232 features for each instance of syncope and non-syncope with different contributions to the classification task. A bar graph depicting the contribution of each feature for first twenty features is shown in Figure 9. It can be inferred from Figure 9 that the maximal value of normalized low frequency diastolic blood pressure (dBPS_LFnu_max) is the feature that contributes the most, followed by the maximal normalized low-frequency RR Interval of HRStats (HRS_RRI_LFnu_max) and the mean of the normalized high-frequency diastolic blood pressure (dBPS_HFnu_mean) in the same order.

#### 3.4.2. Train–Test–Split Evaluation

In the train–test–split evaluation method, the entire dataset is separated into two segments for the purpose of training and then testing the model. This is an important step in which the training set data correspond to the known values of syncope and non-syncope as an attribute, which are tested for the model’s predictions for the test set data. In this work, the entire dataset of 687 patients was divided into the ratio of 80:20, respectively, for training and testing, which is standard practice when using this method of evaluation and has been validated experimentally. This means that 80 percent of the dataset is utilized for training the models, while the remaining 20 percent is used for testing them. This ensures that the test set is neither too small nor too big.

The results derived by the SVM, KNN and SGD-based classification models using train–test–split evaluation in the form of a confusion matrix are shown in Table 7.

Based on the elements of the confusion matrix, a measure of the performance indices of accuracy, *Precision*, *Recall*, *F1-Score* and *AUC-ROC* is presented in Table 8.

It can be inferred from Table 8 that the SVM-based classification model is able to classify the patients with syncope with 97.82% of accuracy which is much higher than the SGD and KNN based classification reporting accuracy of 87.68% and 84.05%, respectively. Similarly, the precision reported by SVM-based model is 98.23% which is again significantly better than precision reported by KNN and SGD being 92.38% and 91.81%, respectively. SVM-based model reports better recall at 99.10% in comparison with 92.66% and 87.38% reported by SGD and KNN, respectively. The *F1-Score* reported by SVM-based model is 98.66% which is again higher than 92.23% and 89.81% as reported by SGD and KNN. Finally, SVM -based model reports much higher *AUC-ROC* of 98.71% in comparison with 90.56% and 83.66% as reported by SGD and KNN. Considering these results, it can be concluded that the SVM-based model is able to perform classification reasonably well reporting superior results for all the considered performance indices as compared to peers.

#### 3.4.3. K-Fold Cross-Validation Evaluation

The K-fold cross-validation is an iterative method used to evaluate the performance of the machine learning model especially when the data sample size is limited. The method evaluates the generalizability of a machine learning model by estimating their performance on unseen or new data with lower bias. Without the cross-validation we only have the assessment of the model over training data that would not provide evaluation of model over new dataset. Thus, after the model has been run using train–test–split, K-fold cross-validation method validates the results.

**Figure 10:**
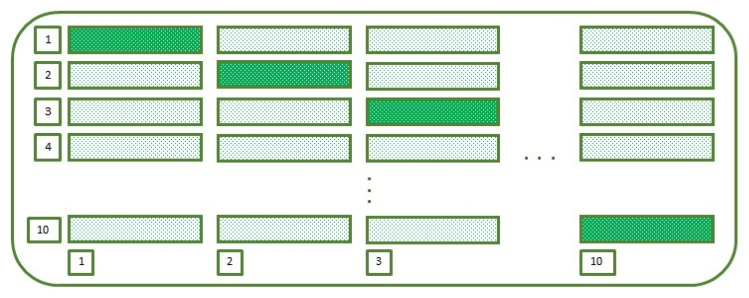
K-fold Cross-Validation Evaluation with K = 10.

In the present work, using K-fold cross-validation, the dataset is divided into K = 10 disjoint sets as shown in Figure 10. The value of K has been chosen carefully through experimentation. The dotted bars represent the subsets of data that have been iteratively used for training the model while the crisscross bar represents the data that have been used for testing it. The process iteratively runs ten times with different sets of data for training and testing purposes.

Using K-fold cross-validation evaluation for ten runs, the performance indices of *accuracy*, *precision*, *recall*, *F1-Score* and *AUC-ROC* were observed for the SVM, KNN and SGD-based models separately. To provide a comprehensive look into the performance of the models, the statistical parameters of results viz. minimal values (Min), maximal values (Max), the number of times the maximum value is attained (No. Of Max), mean values (Mean) and standard deviation (SD) across the tenfold validation of the model were observed and are presented in Table 9.

From Table 9, considering the Mean of each performance indices across the running of the tenfold validation, it can be seen that the SVM model classifies the instances of syncope and non-syncope fairly well, with at least 97.52% *accuracy*, 91.23% *precision*, 92.17% *recall*, 91.39% *F1-Score* and 94.90% *AUC-ROC*. It can be inferred from Table 9 that the SVM-based model is computing results with significantly better accuracy for all statistical parameters of Min, Max, No of Max, Mean and SD in comparison with the SGD and KNN-based models. The case for precision is similar, as the SVM-based model performs better in all categories except for for No of Max, which is higher in KNN. For the remaining indices of *recall*, *F1-Score* and *AUC-ROC*, it can be seen that SVM-based model is performing significantly better than the SGD and KNN-based models for all the considered statistical parameters. Therefore, it can be concluded that for the K-fold cross-validation evaluation of the models, similarly to test–train–split evaluation, the SVM-based model again outperforms its peers on all the performance indices of *accuracy*, *precision*, *recall*, *F1-Score* and *AUC-ROC*. It is to be noted that for the SVM-based model, the maximal value of one for all the performance indices has been attained at least once across the tenfold validation. Additionally, the model computes the highest value four times for precision and recall, two times for *F1-Score* and once each for accuracy and *AUC-ROC*. The reported SD for the SVM-based model proves that the dispersion into the values of indices in relation to their Mean are significantly low, which indicates that the SVM-based model is performing well even for unseen data.

## 4. Limitations and Future Work

In this paper we examined only the robustness of our model against all patients who developed presyncope and those that did not. However, we are aware that different disease conditions, along with polypharmacy, may contribute towards differences in the data, including those related to the ECG. While this comparison was not the focus of the study, this important aspect should definitely be studied in the future, especially across diseases [35] and in older persons [24,36,37,38]. We have previously reported that there are indeed different cardiovascular patterns during graded orthostatic-loading-induced presyncope, albeit in healthy participants [39].

The authors look forward to evaluating the performance of the model being assessed over a significantly large dataset containing the records of thousands of patients. To take the work forward, we intend to examine in detail the features of the ECG and BP to determine whether they can have a predictive value on their own. In this work, the model was created by the application of algorithms to the data, which does not provide much information about the variables and their combinations used in reaching the decisions. To address the same, we are separately working to add more understanding into the decision making of the model by recalling comprehensibility or interpretability. While it is important to assess the generalizability of the data, which is often difficult due to interindividual differences in hemodynamic responses and time to collapse [18], future studies should be carried out to assess the effects of sex [40,41,42], season [43] and/or across the races on the reproducibility of the data obtained.

## 5. Conclusions

For patients having LOC or near LOC, establishing the true form of syncope forms a crucial part for the treatment process. This work differentiates patients who do or do not have induction of syncope and non-syncope based on their physiological indicators by measuring continuous and non-invasive beat-to-beat BP and HR. The machine learning algorithms of SVM, KNN and SGD were employed using the data collected in HUT tests under clinical settings for syncope classification. The models were evaluated for their performance using the train–test–split evaluation mechanism and further validated using K-fold cross-validation. The statistical values-based performance indices observed in the results using both train–test–split evaluation and K-fold cross-validation lead us to the conclusion that the SVM-based model can differentiate between syncope and non-syncope events in a significantly more efficient manner than the KNN and SGD-based models. Therefore, the SVM-based model provides an alternative to the existing diagnostic process and proves the efficiency of using machine learning methods over healthcare data, paving the way for its applicability in the real diagnostic mechanisms used for proactive syncope prediction.

## Figures and Tables

**Table 1:** Age Group and Gender Distribution of Patients having Syncope.

Age Group	Gender	Numbers	Age Group	Gender	Numbers
0–15	M	01	55–65	M	07
	F	02		F	13
15–25	M	01	65–75	M	14
	F	04		F	09
25–35	M	02	75–85	M	03
	F	02		F	11
35–45	M	06	85–95	M	02
	F	03		F	01
45–55	M	07	Total	M	43
	F	08		F	53

**Table 2:** Physiological Indicators of Patients Collected Using HUT Test.

Beatstats			
Acronym	Definition	Equations	Units
HR	Heart Rate	Primitive	Beats/ min
SV	Stroke Volume	Primitive	Litre/beat
CO	Cardiac Output	SV_[l/beat]_ × HR_[bpm]_	Litre/min
CI	Cardiac Input	CO_[l/min]_/Body Surface Area_[m^2^]_	Litre/min/m^2^
SI	Stroke Index	SV_[l/beat]_/Body Surface Area_[m_^2^_]_ × 1000	Ml/beat/m^2^
RRI	RR-Interval	Primitive	Seconds
TPR	Total Peripheral Resistance	Primitive	Pa·sec/m^3^
TPRI	Total Peripheral Resistance Index	Primitive	Pa·sec/m^5^
dBP	Diastolic Blood Pressure	Primitive	mmHg
mBp	Mean Blood Pressure	(2/3) × dBP_[mmHg]_ + (1/3) × sBP_[mmHg]_	mmHg
sBP	Systolic Blood Pressure	Primitive	mmHg
Cardiacbeatstats			
ACI	Acceleration Index	Primitive	m/s^2^
CI	Cardiac Input	CO_[l/min]_/Body Surface Area_[m^2^]_	Litre/min/m^2^
EDI	End-Diastolic Index	Primitive	
HR	Heart Rate	Primitive	Beats/ min
IC	Index of Contractility	Primitive	Seconds
LVET	Left VentricularPrimitiveEjection Time	Primitive	Milliseconds
LVWI	Left Ventricular Stroke Work Index	SI_[ml/beat/m^2^]_ × (LVSP_[mmHg]_—LVEDP_[mmHg]_).	Pa.ml/beat/m^2^
SI	Stroke Index	SV_[l/beat]_/Body Surface Area_[m_^2^_]_ × 1000	Ml/beat/m^2^
TFC	Thoracic Fluid Content	Primitive	Litre
TPRI	Total Peripheral Resistance Index	Primitive	Pa·sec/m^5^
dBP	Diastolic Blood Pressure	Primitive	mmHg
mBp	Mean Blood Pressure	(2/3) × dBP_[mmHg]_ + (1/3) × sBP_[mmHg]_	mmHg
sBP	Systolic Blood Pressure	Primitive	mmHg
HRVstats			
HF_RRI	High-Frequency RR Interval	Primitive	Hz
HFnu_RRI	Normalized High-Frequency RR Interval	HF_RRI/(HF_RRI + LF_RRI + VLF_RRI)	
LF_HF	Difference Between Low and High Frequency of RR Interval	HF_RRI ~ LF_RRI	Hz
LF_HF_RRI	The ratio of Low and High Frequency of RR Interval	LF_RRI/HF_RRI	
LF_RRI	Low-Frequency RR Interval	Primitive	Hz
LFnu_RRI	Normalized Low-Frequency RR Interval	LF_RRI/(HF_RRI +LF_RRI + VLF_RRI)	
PSD_RRI	Power Spectral Density of RR Interval	Primitive	W/Hz
VLF_RRI	Very Low Frequency of RR Interval	Primitive	Hz
dBPVstats			
HF_dBP	High-Frequency dBP	Primitive	Hz
HFnu_dBP	Normalised High-Frequency dBP	HF_dBP/(HF_dBP+ LF_dBP + VLF_dBP)	
LF_HF	Difference Between Low and High Frequency of dBP	HF_dBP ~ LF_dBP	Hz
LF_HF_dBP	Ratio of Low and High Frequency of dBP	LF_dBP/HF_dBP	
LF_dBP	Low-Frequency dBP	Primitive	Hz
LFnu_dBP	Normalised Low-Frequency dBP	LF_dBP/(HF_dBP + LF_dBP + VLF_dBP)	
PSD_dBP	Power Spectral Density of dBP	Primitive	W/Hz
VLF_dBP	Very Low Frequency of dBP	Primitive	Hz
sBPVstats			
HF_sBP	High-Frequency sBP	Primitive	Hz
HFnu_sBP	Normalised High-Frequency sBP	HF_sBP/(HF_sBP +LF_sBP + VLF_sBP)	
LF_HF	Difference Between Low and High Frequency of sBP	HF_sBP ~ LF_sBP	Hz
LF_HF_sBP	Ratio of Low and High Frequency of sBP	LF_sBP/HF_sBP	
LF_sBP	Low-Frequency sBP	Primitive	Hz
LFnu_sBP	Normalised Low-Frequency sBP	LF_sBP/(HF_sBP+ LF_sBP + VLF_sBP)	
PSD_sBP	Power Spectral Density of sBP	Primitive	W/Hz
VLF_sBP	Very Low Frequency of sBP	Primitive	Hz

**Table 3:** Variance Explained by Cumulative PCs.

First PC	20.17%
First two PCs	31.95%
First three PCs	41.41%
First ten PCs	68.24%
First twenty PCs	83.51%
First thirty PCs	90.93%
First forty PCs	94.70%
First fifty PCs	96.71%

**Table 4:** Confusion Matrix.

*C_00_*	*C_01_*
*C_10_*	*C_11_*

*C_00_* denotes the count of true negative data points; *C_01_* denotes the count of false-positive data points; *C_10_* denotes the count of false-negative data points; *C_11_* denotes the count of true positive data points.

**Table 5:** System Specifications.

Hardware Specifications		Software Specifications	
Processor	Core i5	OS	64-bit Windows 10
Processor Clock Speed	1.8 GHz	Scikit learn	0.20.3
Number of Cores	4	Pandas	0.23.4
RAM	8GB	Numpy	1.14.3
Cache Memory	6 MB	Matplotlib	3.0.2
Processor Architecture	64 bit	Seaborn	0.11.1
Processor Variant	8265U	Imblearn	0.00

**Table 6:** Model Parameters.

SVM Parameters							
Parameter	Value	Parameter	Value	Parameter	Value	Parameter	Value
C	2	kernal	linear	degree	3	gamma	auto
coef0	0.0	shrinking	True	probability	False	tol	0.001
cache_ size	200	class_ weight	None	verbose	False	max_iter	−1
decision_ function_ shape	ovr	break_ties	False	random_ state	None		
SGD Parameters							
Parameter	Value	Parameter	Value	Parameter	Value	Parameter	Value
loss	log	penalty	elasticnet	Alpha	0.0001	l1_ratio	0.15
fit_ intercept	true	max_iter	75	Tol	0.001	shuffle	True
verbose	0	epsilon	0.1	n_jobs	None	random_ state	0
learning_ rate	optimal	eta0	0.0	power_t	0.5	early_ stopping	False
validation_ fraction	0.1	n_iter_ no_change	5	class_ weight	None	warm_start	False
KNN Parameters							
Parameter	Value	Parameter	Value	Parameter	Value	Parameter	Value
n_neighbor	5	weight	uniform	algorithm	auto	leaf_ size	30
p	2	metric	minkowski	metric_ param	None	n_jobs	None

**Table 7:** Elements of Confusion Matrix.

Elements	TP	FP	FN	TN
SVM	111	02	01	24
KNN	97	08	14	19
SGD	101	09	08	20

**Table 8:** Results of Train–Test–Split Evaluation.

Measures	SVM	KNN	SGD
*Accuracy*	0.9782608	0.8405797	0.876812
*Precision*	0.9823008	0.923809	0.918182
*Recall*	0.9910714	0.8738738	0.926606
*F1-Score*	0.9866666	0.8981474	0.922375
*AUC-ROC*	0.987123	0.8366731	0.905619

**Table 9:** Result Statistics of K-fold Cross-validation (K = 10).

Measures		Min	Max	No. of Max	Mean	SD
*Accuracy*	SVM	0.955882	1.00	1	0.975256	0.013813
	KNN	0.855073	0.956521	0	0.908299	0.031193
	SGD	0.594203	0.971014	0	0.83241	0.14894
*Precision*	SVM	0.75	1.00	4	0.912387	0.092426
	KNN	0.50	1.00	6	0.917188	0.155584
	SGD	0.5	0.857143	0	0.671813	0.125704
*Recall*	SVM	0.80	1.00	4	0.921715	0.081782
	KNN	0.20	0.70	0	0.434395	0.174226
	SGD	0.496410	1.00	2	0.778064	0.194074
*F1-Score*	SVM	0.80	1.00	2	0.913957	0.069245
	KNN	0.333333	0.823529	0	0.565863	0.173928
	SGD	0.503737	0.923077	0	0.715369	0.146808
*AUC-ROC*	SVM	0.891379	1.00	1	0.949	0.038459
	KNN	0.60	0.85	0	0.713385	0.086626
	SGD	0.366071	0.984127	0	0.667223	0.267143

## Data Availability

The data in this study are readily available upon reasonable request to the corresponding author.
